# Clinicopathological Evaluation of Cholesterolosis in Cholecystitis: Histopathological Patterns and Metabolic Correlates

**DOI:** 10.3390/jcm14186500

**Published:** 2025-09-15

**Authors:** Damla Gül Fındık, Erhan Şahin, Özlem Türelik

**Affiliations:** 1 Department of Histology and Embryology, Faculty of Medicine, Bilecik Şeyh Edebali University, 11230 Bilecik, Turkey; erhan.sahin@bilecik.edu.tr; 2 Department of Pathology, Faculty of Medicine, Bilecik Şeyh Edebali University, 11230 Bilecik, Turkey; ozlem.turelik@bilecik.edu.tr

**Keywords:** blood glucose, cholecystitis, diabetes mellitus, gallbladder diseases, inflammation

## Abstract

**Background/Objectives**: Cholesterolosis is a relatively common, yet often incidental, histopathological finding in cholecystectomy specimens, frequently associated with chronic cholecystitis. Although its clinical significance remains unclear, possible associations with glycemic regulation have been proposed. This study aimed to examine the relationship between cholesterolosis, glycemic parameters, and inflammatory markers in patients with chronic cholecystitis. **Methods**: We retrospectively analyzed gallbladder specimens from patients who underwent cholecystectomy for chronic cholecystitis between 2014 and 2023. Histopathology assessed cholesterolosis presence, distribution, and inflammatory features. Patients were grouped according to diabetes status (diabetic vs. non-diabetic) as well as the presence or absence of cholesterolosis. Demographic data, gallstone status, one-year mean fasting glucose, HbA1c levels, and hematological/inflammatory indices were compared between groups. **Results**: Among diabetic patients, those with cholesterolosis had significantly lower fasting glucose than those without cholesterolosis (129.04 ± 28.02 vs. 158.41 ± 54.23 mg/dL, *p* < 0.05). The presence of cholesterolosis was not significantly associated with diabetes status (*p* > 0.05). Inflammatory indices, including neutrophil count, did not differ significantly between groups (*p* > 0.05), although fasting glucose correlated positively with neutrophil count (*r* = 0.167, *p* < 0.05). Gallstones were less frequent in cholesterolosis cases compared with non-cholesterolosis cases (59.4% vs. 75%, *p* < 0.05), suggesting a distinct pathophysiology. **Conclusions:** This is the first study to assess one-year glycemic profiles in diabetic and non-diabetic patients with and without cholesterolosis. The findings suggest that cholesterolosis may occur independently of poor glycemic control and systemic inflammation, supporting the concept of a distinct underlying mechanism.

## 1. Introduction

Cholesterolosis is defined by the accumulation of lipid-laden macrophages in the subepithelial region of the lamina propria of the gallbladder. It may present in focal, diffuse, or polypoid forms and is detected in approximately 20% of cholecystectomy specimens, particularly in cases of chronic cholecystitis [1,2]. The condition is thought to result from the deposition of cholesterol esters due to increased cholesterol ester synthesis in the gallbladder mucosa, which may be driven by dysregulation of intracellular cholesterol homeostasis [2]. Despite its prevalence, the clinicopathological significance of cholesterolosis remains unclear, though possible associations with metabolic disorders have been suggested [2,3,4].

In gallbladder epithelial cells, intracellular cholesterol balance depends on the coordinated actions of acyl-CoA cholesterol acyltransferase 1 (ACAT1), neutral cholesteryl ester hydrolase 1 (NCEH1), and the ATP-binding cassette transporter A1 (ABCA1), which collectively regulate esterification, hydrolysis, and efflux processes [5]. Interestingly, ACAT1 expression is reduced in the adipose tissue of obese individuals with type 2 diabetes, linking cholesterolosis to systemic metabolic disturbances [6]. However, its etiology appears distinct from inflammatory conditions associated with acute or chronic cholecystitis, and it is not considered a premalignant lesion [7].

Previous studies on cholesterolosis and metabolic parameters yielded conflicting findings. While some reported no correlation with serum glucose, others suggested a link between fasting blood glucose (FBG) and cholesterolosis [2,8,9,10]. A major limitation has been the lack of subgrouping according to diabetes status, which may explain inconsistencies. To address this gap, we retrospectively analyzed 253 patients with calculous or acalculous chronic cholecystitis diagnosed between 2014 and 2023. One-year mean HbA1c and FBG values were evaluated to determine the impact of glycemic control on cholesterolosis in diabetic, prediabetic, and normoglycemic patients, along with the potential modifying effects of gallstone status and systemic inflammatory parameters.

This study provides a comprehensive assessment of how variations in glycemic control influence the occurrence of cholesterolosis alongside chronic cholecystitis. Systemic inflammatory status was also evaluated using hematological inflammatory indices. We hypothesize that the relationship between cholesterolosis and glycemic control differs according to diabetes status. By investigating these relationships, our findings offer new insights into the potential mechanisms linking glycemic disturbances to gallbladder pathology, thereby contributing to a more comprehensive understanding of cholesterolosis pathogenesis. These insights may inform future diagnostic and therapeutic strategies in chronic gallbladder disease.

## 2. Materials and Methods

### 2.1. Subjects and Inclusion/Exclusion Criteria

This study is a retrospective analysis based on the histopathological records of patients diagnosed with chronic calculous or acalculous cholecystitis, who underwent cholecystectomy at the hospital between January 2014 and April 2023. We specifically included patients with documented HbA1c and FBG levels, measured within the year preceding surgery, ensuring a uniform timeframe for evaluating glycemic control. Cases were categorized according to the presence of cholesterolosis and their diabetes status (diabetic, prediabetic vs. non-diabetic). Given that antidiabetic medication can substantially influence FBG and HbA1c, patients with a clinical diagnosis of diabetes were not reclassified according to laboratory cut-offs. Instead, diabetes was defined based on the established clinical diagnosis, while only non-diabetic individuals were categorized as normal (FBG < 100 mg/dL) or pre-diabetic (FBG 100–125 mg/dL). In addition to diabetes and cholesterolosis status, demographic data including age and gender, as well as clinical parameters such as the presence of gallstones, were documented for detailed statistical analysis. This comprehensive data collection was aimed at examining any correlations between these factors and the development of cholesterolosis in patients with chronic cholecystitis.

To minimize confounding variables and ensure that the results were not skewed by other underlying conditions, patients with additional pathologies such as gallbladder cancer, metaplasia, dysplasia, or adenomyomatous hyperplasia were excluded from the analysis. These exclusion criteria were applied to isolate the effects of cholesterolosis on glycemic control, minimizing potential confounding factors. To ensure consistency, patients with discordant results (diabetic-range FBG without a clinical diagnosis) were excluded from subgroup analyses (*n* = 9). Ethical approval was obtained from the Ethics Committee of Bilecik University (approval number: 2023/4-6). All methods were conducted in accordance with the ethical standards of the institutional research committee and with the Declaration of Helsinki.

### 2.2. Histopathological Examination

Gallbladder tissue samples were collected immediately after surgical removal and fixed in 10% neutral buffered formaldehyde to preserve the tissue integrity. After fixation, the samples underwent standard histopathological processing, which involved dehydration, clearing, and paraffin embedding. Following embedding, five-micrometer sections were cut from the paraffin blocks and mounted on standard glass slides for further examination.

The slides were deparaffinized using xylene, followed by a rehydration process through a graded ethanol series (100%, 90%, 80%, and 70%). Once rehydrated, the tissue sections were stained with hematoxylin and eosin (H&E), a standard procedure that allows for clear visualization of tissue structures and abnormalities. After staining, the slides were mounted with Entellan to preserve the stained tissue. Histopathological evaluation of the slides was performed under an Olympus CX23 brightfield microscope (CX23, Olympus, Tokyo, Japan) equipped with an Olympus EP50 camera (1920 × 1080 pixels; EP50, Olympus, Tokyo, Japan), enabling detailed imaging of the tissue and identification of key pathological features associated with cholesterolosis, such as the accumulation of lipids in the lamina propria and the presence of gallbladder polyps.

### 2.3. Statistical Analysis

All statistical analyses were performed using IBM SPSS Statistics software (version 26.0, IBM Corp, Armonk, NY, USA). A *p*-value of less than 0.05 was considered statistically significant for all tests. The Kolmogorov–Smirnov test was used to assess the normality of continuous variables. Nonparametric continuous variables were compared using the Mann–Whitney U test, while parametric continuous variables were analyzed with the independent sample *t*-test. Categorical variables, including the presence of gallstones and cholesterolosis, were evaluated using the Chi-square test to identify significant associations between categorical data. Spearman’s correlation analysis was employed to evaluate the relationships between continuous variables.

## 3. Results

### 3.1. Histopathological Results

**Figure 1:**
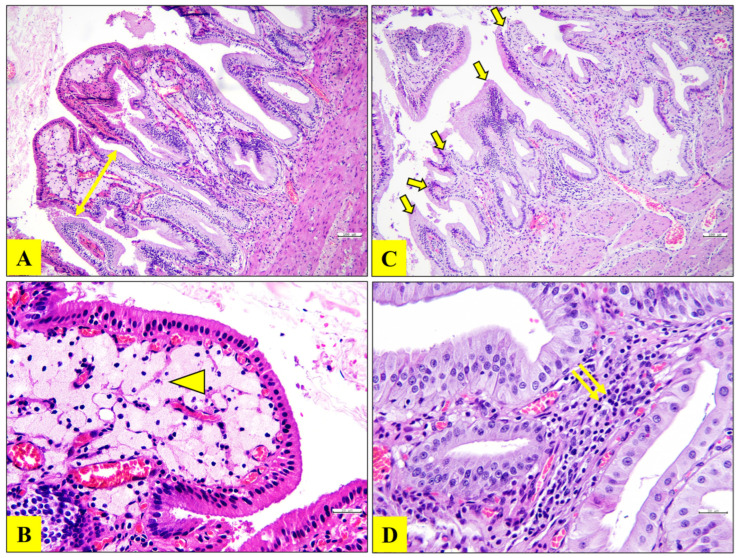
Hematoxylin-eosin staining of patients diagnosed with cholesterolosis (**A**,**B**) and chronic cholecystitis (**C**,**D**). (**A**,**B**) Villus hypertrophy (double-headed arrow) and abundant foamy histiocytes (lipid-loaded) (arrowhead) in the lamina propria of the cholesterolosis group. (**C**,**D**) Villus hyperplasia (thick arrows) and mild mononuclear cell infiltration (double arrow) in the lamina propria of chronic cholecystitis patients. Scale bars: (**A**,**C**) 100 µm, (**B**,**D**) 30 µm.

Histopathological examination of gallbladder specimens revealed distinct features associated with cholesterolosis and chronic cholecystitis. In patients diagnosed with cholesterolosis, villus hypertrophy and an accumulation of lipid-laden foamy histiocytes were prominent within the lamina propria, reflecting excessive cholesterol deposition. The presence of foamy histiocytes is a hallmark histological feature of cholesterolosis. In contrast, gallbladders from patients with chronic cholecystitis but without cholesterolosis exhibited villus hyperplasia accompanied by mild mononuclear cell infiltration in the lamina propria (Figure 1).

### 3.2. Association of Cholesterolosis with Age, Gender, Gallstones, Acute Attack, and Diabetes Mellitus

The mean age of patients with cholesterolosis (53.09 ± 11.2 years) was not significantly different from those without cholesterolosis (53.16 ± 13.96 years) (*p* > 0.05), suggesting that cholesterolosis does not have a strong age-related predisposition within the context of cholecystitis. Likewise, no significant association was observed between cholesterolosis and gender [χ^2^(1, *N* = 253) = 2.36, *p* = 0.125], as both groups were predominantly female (cholesterolosis: 84.1%; non-cholesterolosis: 75%), indicating that gender is not a major determinant in the occurrence of cholesterolosis (Table 1).

Interestingly, gallstones were significantly more prevalent in the non-cholesterolosis group (75%) compared to the cholesterolosis group, a difference that reached statistical significance [χ^2^(1, *N* = 253) = 5.89, *p* = 0.015] (Table 1).

The comparison of acute attack incidence between the cholesterolosis and non-cholesterolosis groups revealed no significant difference (*p* > 0.05). In the cholesterolosis group, 2.9% individuals had an acute attack, while 2.7% individuals in the non-cholesterolosis group experienced an acute attack.

No significant association was found between cholesterolosis and diabetes mellitus [χ^2^(2, *N* = 253) = 1.01, *p* = 0.604], indicating that the presence of diabetes does not appear to be a key factor in cholesterolosis development. These findings support the notion that cholesterolosis is not solely linked to diabetes (Table 1).

### 3.3. Glycemic Parameters in Chronic Cholecystitis Patients

Glycemic parameters did not differ significantly between cholesterolosis and non-cholesterolosis groups across all patients, with comparable mean HbA1c (6.35 ± 1.33% vs. 6.10 ± 0.94%) and FBG levels (129.58 ± 46.75 vs. 120.85 ± 31.38 mg/dL) (*p* > 0.05) (Table 1).

**Figure 2:**
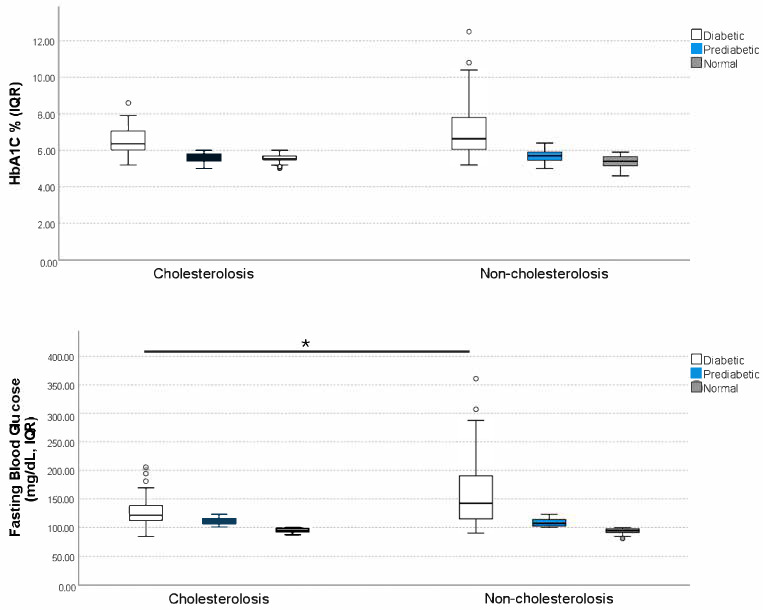
HbA1c and fasting blood glucose (FBG) levels in diabetic and non-diabetic chronic cholecystitis patients (diagnosed as cholesterolosis and non-cholesterolosis). Mann–Whitney U test; IQR: interquartile range; whiskers show min–max; outliers as points; statistical significance: * (*p* < 0.05).

In diabetic patients with chronic cholecystitis, a significant difference was observed in FBG levels between cholesterolosis and non-cholesterolosis groups. Diabetic patients with cholesterolosis had significantly lower FBG levels (129.04 ± 28.02 mg/dL) compared to those without cholesterolosis (158.41 ± 54.23 mg/dL) (*p* < 0.05). This finding suggests that better glycemic control, reflected by lower FBG levels, may be associated with the presence of cholesterolosis in diabetic individuals (Table 2) (Figure 2).

Although HbA1c levels were slightly higher in the non-cholesterolosis diabetic group (7.12 ± 1.47%) compared to the cholesterolosis diabetic group (6.67 ± 1.03%), this difference was not statistically significant (*p* > 0.05) (Table 2) (Figure 2).

Among non-diabetic patients, glycemic parameters (HbA1c and FBG levels) were similar between cholesterolosis and non-cholesterolosis groups. The mean HbA1c values did not differ significantly between cholesterolosis and non-cholesterolosis groups in pre-diabetic individuals (5.60 ± 0.28% vs. 5.71 ± 0.35%, *p* > 0.05) or in normoglycemic individuals (5.51 ± 0.27% vs. 5.35 ± 0.32%, *p* > 0.05). Similarly, FBG levels showed no significant differences in the pre-diabetic group (111.07 ± 6.03 vs. 108.54 ± 6.73 mg/dL, *p* > 0.05) or in the normoglycemic group (94.45 ± 4.31 vs. 93.87 ± 4.87 mg/dL, *p* > 0.05) (Table 2, Figure 2). These results indicate that glycemic control in non-diabetic individuals does not appear to significantly influence the development of cholesterolosis, suggesting that other metabolic or physiological factors may play a more prominent role in its pathogenesis.

### 3.4. Hematological Parameters in Chronic Cholecystitis Patients

The analysis of hematological parameters, including neutrophil, lymphocyte, platelet, and monocyte counts as well as inflammatory indices (NLR: Neutrophil-to-Lymphocyte Ratio, LMR: Lymphocyte-to-Monocyte Ratio, PLR: Platelet-to-Lymphocyte Ratio, SII: Systemic Immune-Inflammation Index), revealed no significant differences between the cholesterolosis and non-cholesterolosis groups when all patients were considered, regardless of their diabetic status. The mean values for neutrophils (4.71 ± 2.04 vs. 4.98 ± 2.48), lymphocytes (2.34 ± 0.84 vs. 2.44 ± 0.75), platelets (268.26 ± 71.83 vs. 266.47 ± 67.87), and monocytes (0.48 ± 0.19 vs. 0.56 ± 0.31) were comparable between the cholesterolosis and non-cholesterolosis groups, with no statistically significant differences observed (*p* > 0.05). Similarly, inflammatory indices such as NLR (2.37 ± 0.16 vs. 2.34 ± 0.24), LMR (5.68 ± 0.38 vs. 5.18 ± 0.29), PLR (129.29 ± 5.05 vs. 119.74 ± 5.78), and SII (642.76 ± 48.09 vs. 627.64 ± 69.21) did not show significant differences between the two groups (*p* > 0.05) (Table 1).

When examining the data by diabetic status, no significant differences in hematological parameters were found between the cholesterolosis and non-cholesterolosis groups among diabetic patients. In this subgroup, the mean neutrophil (4.99 ± 3.06 vs. 5.08 ± 2.43), lymphocyte (2.50 ± 0.95 vs. 2.34 ± 0.96), platelet (280.77 ± 64.56 vs. 266.47 ± 83.54), and monocyte (0.60 ± 0.45 vs. 0.50 ± 0.19) counts were similar between the two groups (*p* > 0.05). Additionally, no significant differences were observed in inflammatory indices such as NLR (2.33 ± 0.44 vs. 2.79 ± 0.30), LMR (5.71 ± 0.68 vs. 5.13 ± 0.26), PLR (125.08 ± 7.97 vs. 133.82 ± 8.59), and SII (655.31 ± 119.69 vs. 733.50 ± 86.82) (*p* > 0.05) (Table 2).

In non-diabetic patients, a significant difference was observed in lymphocyte count within the pre-diabetic subgroup, with the cholesterolosis group showing higher values (2.58 ± 0.78) compared to the non-cholesterolosis group (2.30 ± 0.68) (*p* < 0.05). No significant differences were found for other hematological parameters, including neutrophil count (4.98 ± 1.65 vs. 4.73 ± 1.87), platelet count (282.92 ± 77.62 vs. 260.55 ± 55.72), and monocyte count (0.52 ± 0.13 vs. 0.48), as well as inflammatory indices including NLR (2.31 ± 0.41 vs. 2.14 ± 0.13), LMR (5.15 ± 0.35 vs. 6.36 ± 1.05), PLR (125.17 ± 13.50 vs. 123.16 ± 5.90), and SII (686.16 ± 136.74 vs. 580.62 ± 41.58) (*p* > 0.05). Similarly, in the normal non-diabetic subgroup, no significant differences were observed between cholesterolosis and non-cholesterolosis groups for lymphocyte count (2.37 ± 0.55 vs. 2.33 ± 0.53), neutrophil count (4.17 ± 0.90 vs. 3.97 ± 1.38), platelet count (253.15 ± 56.03 vs. 262.83 ± 56.47), and monocyte count (0.50 ± 0.18 vs. 0.46 ± 0.17), or for inflammatory indices including NLR (1.83 ± 0.15 vs. 1.79 ± 0.11), LMR (5.20 ± 0.60 vs. 5.41 ± 0.34), PLR (113.64 ± 13.26 vs. 117.66 ± 6.02), and SII (474.60 ± 58.23 vs. 474.42 ± 42.05) (*p* > 0.05) (Table 2).

### 3.5. Correlation of Hematological Parameters and Inflammatory Indices with Glycemic Parameter

Correlation analysis between FBG levels and various hematological parameters and inflammatory indices was performed for all patients, as well as separately for diabetic and non-diabetic subgroups.

In the analysis of all patients, a positive correlation was found between FBG and neutrophil count (*r* = 0.167 *p* < 0.05), indicating that as FBG levels increase, neutrophil counts tend to rise. However, no significant correlation was observed between FBG and other hematological parameters, such as lymphocyte count, platelet count, and monocyte count. Inflammatory indices, including NLR, LMR, PLR, and SII, showed no significant correlation with FBG (*p* > 0.05) (Table 3).

Correlation analysis between FBG and hematological parameters or inflammatory indices revealed no significant associations in any subgroup. In diabetic patients, FBG showed no significant correlation with neutrophil count (*r* = 0.136, *p* > 0.05). Likewise, in pre-diabetic and normoglycemic non-diabetic individuals, FBG was not significantly correlated with neutrophil count (*r* = 0.176, *p* > 0.05 and *r* = −0.061, *p* > 0.05, respectively) (Table 3).

## 4. Discussion

The relationship between cholesterolosis and glycemic parameters, particularly FBG and HbA1c, remains an area of ongoing investigation with inconsistent findings. While previous studies have explored the metabolic implications of cholesterolosis, the precise link between this condition and glycemic control remains unclear. Although serum lipid levels have been extensively studied in relation to cholesterolosis, the evaluation of glucose parameters has been less explored [11]. In this study, we examined the one-year mean HbA1c and FBG levels in patients with chronic cholecystitis, considering their cholesterolosis and diabetes status to better understand this relationship.

Our findings align with prior research, such as Yoon et al., who reported significant glucose variations in cholecystitis patients based on cholesterolosis status [12]. Similarly, Dairi et al. observed lower serum glucose levels (<10 mg/dL) in cholecystectomy specimens with cholesterolosis [2]. In our study, diabetic patients with chronic cholecystitis and cholesterolosis exhibited a notable reduction (>25 mg/dL) in FBG levels over one year. These results suggest that cholesterolosis may not exacerbate glycemic dysregulation in diabetic patients but rather may be associated with improved glycemic control.

In the context of metabolic syndrome, abnormal lipid profiles—particularly elevated cholesterol levels—are well-established risk factors for gallstone formation and other gallbladder-related diseases. Obesity, a key component of metabolic syndrome, further exacerbates gallbladder dysfunction by promoting fat accumulation, inflammation, and dysmotility [13]. Although improved glycemic control mitigates some metabolic risks, persistent dyslipidemia may still contribute to cholesterolosis development. Consequently, despite improved glycemic control, the risk of developing conditions such as cholesterolosis and cholecystitis may persist if other metabolic disturbances remain unaddressed. Additionally, chronic hyperglycemia has been implicated in immune dysfunction, leading to increased inflammation through mechanisms such as complement protein glycosylation and neutrophil dysfunction. This inflammatory state in diabetic patients could play a role in cholecystitis pathogenesis [14]. Dolanbay et al. reported an increased risk of acalculous cholecystitis in diabetic patients, supporting this hypothesis [15]. In our study, we found a weak positive correlation between FBG and neutrophil count, but the presence of cholesterolosis did not significantly affect hematological parameters or inflammatory indices. Importantly, this pattern persisted across both prediabetic and normoglycemic non-diabetic patients, suggesting that cholesterolosis alone is unlikely to drive systemic inflammation. These findings are consistent with prior reports [6,16]. The results indicate that, while metabolic and glycemic abnormalities may predispose one to gallbladder inflammation, cholesterol accumulation appears to be a localized process without a major effect on systemic inflammatory markers.

Prediabetic glycemic levels are defined as HbA1c between 5.7 and 6.4% and FBG between 100 and 125 mg/dL [17]. In this study, the inclusion of a prediabetic group allowed us to more comprehensively evaluate the relationship between glycemic control and cholesterolosis. Among non-diabetic patients, no significant differences in HbA1c (prediabetic: 5.60 ± 0.28% vs. 5.71 ± 0.35%; normal: 5.51 ± 0.27% vs. 5.35 ± 0.32%) or FBG (prediabetic: 111.07 ± 6.03 vs. 108.54 ± 6.73 mg/dL; normal: 94.45 ± 4.31 vs. 93.87 ± 4.87 mg/dL) were observed between cholesterolosis and non-cholesterolosis groups, regardless of whether patients were prediabetic or normoglycemic. These findings suggest that elevated glycemic markers, even within the prediabetic range, are not directly associated with the presence of cholesterolosis. Among the study population, the prevalence of diabetes, prediabetes, and normoglycemia was similar between the cholesterolosis and non-cholesterolosis groups (diabetic: 43.5% vs. 50.5%; prediabetic: 37.7% vs. 32.6%; normal: 18.8% vs. 16.8%), further supporting that systemic elevated glycemic levels alone are unlikely to be the primary driver of cholesterol accumulation in the gallbladder.

The potential influence of diabetes medications on gallbladder pathology is another critical consideration. Some antidiabetic agents, including dipeptidyl peptidase 4 inhibitors (DPP-4i) and glucagon-like peptide-1 receptor agonists (GLP-1 RA), have been associated with biliary diseases [18,19,20,21]. Recent pharmacovigilance data indicate that DPP-4 inhibitors, particularly sitagliptin, and certain GLP-1 RAs, such as semaglutide and liraglutide, may be linked to increased biliary risk [22]. Teneligliptin, a dipeptidyl peptidase 4 inhibitor, has been reported to suppress foam cell formation and ACAT-1 gene expression in macrophages [23]. In an experimental animal model fed with a lithogenic diet, pioglitazone prevented gallstone formation and improved systemic metabolic parameters. Mechanistically, these effects were associated with the downregulation of ACAT2 and Niemann-Pick C1-Like 1 (NPC1L1) in the intestine, alongside modulation of hepatic enzymes involved in cholesterol synthesis and bile acid metabolism [24]. Experimental studies on gallbladder epithelial cells have shown that pioglitazone can promote cholesterol ester hydrolysis and efflux by upregulating ABCA1 and NCEH1 [5]. Future research should explore how these medications affect cholesterolosis development and progression. Most studies have focused on hepatic effects, and the specific molecular mechanisms in the gallbladder remain unclear.

The prevalence of cholesterolosis varies among different cholecystitis subtypes. Mohan et al. reported cholesterolosis in 16% of acalculous chronic cholecystitis and 9.9% of cholelithiasis cases [25]. In our study, cholesterolosis was observed in 37.8% of acalculous and 22.9% of calculous chronic cholecystitis cases. Notably, the lower prevalence of gallstones in the cholesterolosis group (59.4% vs. 75% in the non-cholesterolosis group) suggests that these two entities may involve distinct pathophysiological pathways. While cholesterol gallstones are thought to develop in individuals unable to maintain cholesterol in solution, those who can solubilize cholesterol may instead exhibit increased mucosal uptake, leading to cholesterolosis. This process has been linked to the enhanced activity of acyl-CoA cholesterol esterifying enzymes and subsequent accumulation of cholesterol esters within gallbladder macrophages [1]. A study on monozygotic twins suggests a shared genetic background, yet variations in gallbladder cholesterol deposition appear to be influenced by gallbladder motility, sphincter of Oddi function, and local cholesterol handling. In this study, one twin developed gallstones associated with impaired motility and Oddi hypertonia, while the other exhibited cholesterolosis despite preserved motility. These observations underscore the potential contributions of epigenetic, metabolic, and environmental factors in determining the site of cholesterol deposition [26]. Isoform-specific differences in ACAT activity may provide an additional mechanistic explanation, with ACAT2 particularly implicated in gallstone formation, whereas altered ACAT1-driven macrophage esterification may contribute more prominently to cholesterolosis [27]. These molecular distinctions are further highlighted by MUC3 and MUC5B, which were overexpressed in cholesterol gallstones but remained unchanged in cholesterolosis, supporting the notion of distinct pathogenic mechanisms for gallbladder cholesterol deposition [28].

Demographic factors also play a significant role in gallbladder disease. Dairi et al. reported that 70% of patients in the cholesterolosis group and 81% in the non-cholesterolosis group were female, with mean ages of 46 ± 16.3 years and 54 ± 18.7 years, respectively [2]. Previous studies have similarly identified a female predominance in cholesterolosis cases, with comparable age distributions, consistent with our findings (84.1% female, 53.09 ± 11.2 years in the cholesterolosis group vs. 75% female, 53.16 ± 13.96 years in the non-cholesterolosis group) [14,29,30]. In the current study, age and sex did not appear to have a significant influence on the pathogenesis of cholesterolosis.

Hyperglycemia and hyperinsulinemia are known to stimulate cholesterol ester synthesis in macrophages, contributing to lipid accumulation in these cells [31,32]. This process could theoretically contribute to the development of cholesterolosis in diabetic patients. A 2016 study by Dairi et al. observed a higher rate of comorbid diabetes in patients with cholecystitis, leading to the hypothesis that diabetes might be associated with cholesterolosis [2]. However, our study, which specifically focused on chronic cholecystitis, did not find a significant association between diabetes status and the presence of cholesterolosis. This discrepancy may be attributed to differences in study populations or design, as our study exclusively examined chronic cholecystitis cases, which may present different pathophysiological mechanisms compared to other types of cholecystitis.

One limitation of this study is its retrospective, single-center design. Additionally, insulin levels were not measured in all patients, which limits the ability to comprehensively assess the role of insulin signaling in the development of cholesterolosis. Despite these limitations, the study benefits from a dataset spanning multiple years, providing valuable insight into the relationship between glycemic parameters and cholesterolosis. Future prospective studies with complete metabolic profiling are warranted to clarify these associations.

Our findings indicate that cholesterolosis is not dependent on diabetes status, although in diabetic patients it is associated with improved glycemic control independent of systemic inflammation, suggesting that severe hyperglycemia is not a primary factor in its development. The lower prevalence of gallstones in cholesterolosis patients further supports distinct underlying mechanisms separate from gallstone formation. These results highlight the complex relationship between glycemic regulation and gallbladder pathology and underscore the need for further research to elucidate the metabolic mechanisms of cholesterolosis, which may inform future diagnostic and therapeutic strategies in chronic gallbladder disease.

## Figures and Tables

**Table 1:** Comparison of clinical and hematological parameters between cholesterolosis and non-cholesterolosis groups.

	Cholesterolosis	Non-Cholesterolosis	*p* Value
Age (years)	53.09 ± 11.2	53.16 ± 13.96	0.970 ^a^
Gender			
Male (*n*)	11 (15.9%)	46 (25%)	0.125 ^b^
Female (*n*)	58 (84.1%)	138 (75%)	
Total%	69 (100%)	184 (100%)	
Gallstone			
Positive (*n*)	41 (59.4%)	138 (75%)	* 0.015 ^b^
Negative (*n*)	28 (40.6%)	46 (25%)	
Total%	69 (100%)	184 (100%)	
Acute attack			
Positive (*n*)	2 (2.9%)	5 (2.7%)	1.000 ^b^
Negative (*n*)	67 (97.1%)	179 (97.3%)	
Total%	69 (100%)	184 (100%)	
Diabetes			
Diabetic (*n*)	30 (43.5%)	93 (50.5%)	0.604 ^b^
Prediabetic (*n*)	26 (37.7%)	60 (32.6%)	
Normal (*n*)	13 (18.8%)	31 (16.8%)	
Total%	69 (100%)	184 (100%)	
HbA1c (%)	6.35 ± 1.33	6.10 ± 0.94	0.229 ^c^
FBG (mg/dL)	129.58 ± 46.75	120.85 ± 31.38	0.236 ^c^
Neu (10^3^/μL)	4.71 ± 2.04	4.98 ± 2.48	0.858 ^c^
Lym (10^3^/μL)	2.34 ± 0.84	2.44 ± 0.75	0.098 ^c^
PLT (10^3^/μL)	268.26 ± 71.83	266.47 ± 67.87	0.230 ^c^
Mon (10^3^/μL)	0.48 ± 0.19	0.56 ± 0.31	0.213 ^c^
NLR	2.37 ± 0.16	2.34 ± 0.24	0.279 ^c^
LMR	5.68 ± 0.38	5.18 ± 0.29	0.990 ^c^
PLR	129.29 ± 5.05	119.74 ± 5.78	0.522 ^c^
SII	642.76 ± 48.09	627.64 ± 69.21	0.569 ^c^

Data are shown as mean ± SD, mean ± SEM (ratios) or number and percentage (%). ^a^ Independent sample *t*-test, ^b^ Chi-square test, ^c^ Mann–Whitney U Test. Neu: Neutrophils, Lym: Lymphocytes, PLT: Platelets, Mon: Monocytes, FBG: Fasting blood glucose, NLR: Neutrophil-to-Lymphocyte Ratio, LMR: Lymphocyte-to-Monocyte Ratio, PLR: Platelet-to-Lymphocyte Ratio, SII = Systemic Immune-Inflammation Index (calculated as Platelets × Neutrophils/Lymphocytes), SD: Standard deviation, SEM: Standard error, Statistical significance: * (*p* < 0.05).

**Table 2:** Comparison of clinical and hematological parameters in diabetic and non-diabetic groups.

	**Diabetic**		
	**Cholesterolosis**	**Non-Cholesterolosis**	***p* Value**
HbA1c (%)	6.67 ± 1.03	7.12 ± 1.47	0.263 ^c^
FBG (mg/dL)	129.04 ± 28.02	158.41 ± 54.23	* 0.009 ^c^
Neu (10^3^/μL)	4.99 ± 3.06	5.08 ± 2.43	0.483 ^c^
Lym (10^3^/μL)	2.50 ± 0.95	2.34 ± 0.96	0.483 ^c^
PLT (10^3^/μL)	280.77 ± 64.56	266.47 ± 83.54	0.214 ^c^
Mon (10^3^/μL)	0.60 ± 0.45	0.50 ± 0.19	0.578 ^c^
NLR	2.33 ± 0.44	2.79 ± 0.30	0.281 ^c^
LMR	5.71 ± 0.68	5.13 ± 0.26	0.506 ^c^
PLR	125.08 ± 7.97	133.82 ± 8.59	0.617 ^c^
SII	655.31 ± 119.69	733.50 ± 86.82	0.621 ^c^
	**Pre-diabetic**		
	**Cholesterolosis**	**Non-cholesterolosis**	***p* value**
HbA1c (%)	5.60 ± 0.28	5.71 ± 0.35	0.197 ^c^
FBG (mg/dL)	111.07 ± 6.03	108.54 ± 6.73	0.056 ^c^
Neu (10^3^/μL)	4.98 ± 1.65	4.73 ± 1.87	0.342 ^c^
Lym (10^3^/μL)	2.58 ± 0.78	2.30 ± 0.68	* 0.049 ^c^
PLT (10^3^/μL)	282.92 ± 77.62	260.55 ± 55.72	0.308 ^c^
Mon (10^3^/μL)	0.52 ± 0.13	0.48 ± 0.19	0.147 ^c^
NLR	2.31 ± 0.41	2.14 ± 0.13	0.579 ^c^
LMR	5.15 ± 0.35	6.36 ± 1.05	0.767 ^c^
PLR	125.17 ± 13.50	123.16 ± 5.90	0.397 ^c^
SII	686.16 ± 136.74	580.62 ± 41.58	0.728 ^c^
	**Normal**		
	**Cholesterolosis**	**Non-cholesterolosis**	***p* value**
HbA1c (%)	5.51 ± 0.27	5.35 ± 0.32	0.175 ^c^
FBG (mg/dL)	94.45 ± 4.31	93.87 ± 4.87	0.907 ^c^
Neu (10^3^/μL)	4.17 ± 0.90	3.97 ± 1.38	0.505 ^c^
Lym (10^3^/μL)	2.37 ± 0.55	2.33 ± 0.53	0.886 ^c^
PLT (10^3^/μL)	253.15 ± 56.03	262.83 ± 56.47	0.522 ^c^
Mon (10^3^/μL)	0.50 ± 0.18	0.46 ± 0.17	0.705 ^c^
NLR	1.83 ± 0.15	1.79 ± 0.11	0.824 ^c^
LMR	5.20 ± 0.60	5.41 ± 0.34	0.610 ^c^
PLR	113.64 ± 13.26	117.66 ± 6.02	0.243 ^c^
SII	474.60 ± 58.23	474.42 ± 42.05	1.000 ^c^

Data are shown as mean ± SD, mean ± SEM (ratios), or number and percentage (%). ^c^ Mann–Whitney U Test. Neu: Neutrophils, Lym: Lymphocytes, PLT: Platelets, Mon: Monocytes, FBG: Fasting blood glucose, NLR: Neutrophil-to-Lymphocyte Ratio, LMR: Lymphocyte-to-Monocyte Ratio, PLR: Platelet-to-Lymphocyte Ratio, SII: Systemic Immune-Inflammation Index (calculated as Platelets × Neutrophils/Lymphocytes), SD: Standard deviation, SEM: Standard error, Statistical significance: * (*p* < 0.05).

**Table 3:** Correlation analysis of fasting blood glucose with hematological parameters and inflammatory indices.

		All Cases FBG	Diabetic Cases FBG	Pre-Diabetic Cases FBG	Normal Cases FBG
Neu	*r*	0.167	0.136	0.176	−0.061
	*p*	* 0.008	0.133	0.105	0.698
Lym	*r*	0.015	0.036	0.040	−0.040
	*p*	0.816	0.691	0.716	0.798
PLT	*r*	0.016	−0.018	0.011	−0.165
	*p*	0.805	0.840	0.917	0.290
Mon	*r*	0.085	0.078	0.124	−0.370
	*p*	0.177	0.389	0.254	0.015
NLR	*r*	0.120	0.067	0.072	−0.051
	*p*	0.057	0.464	0.507	0.747
LMR	*r*	−0.040	0.008	−0.058	0.225
	*p*	0.523	0.930	0.599	0.147
PLR	*r*	−0.013	−0.098	−0.048	−0.055
	*p*	0.833	0.283	0.662	0.726
SII	*r*	0.096	0.000	0.090	−0.087
	*p*	0.126	0.998	0.409	0.580

Spearman’s correlation test, Neu: Neutrophils, Lym: Lymphocytes, PLT: Platelets, Mon: Monocytes, FBG: Fasting blood glucose, NLR: Neutrophil-to-Lymphocyte Ratio, LMR: Lymphocyte-to-Monocyte Ratio, PLR: Platelet-to-Lymphocyte Ratio, SII: Systemic Immune-Inflammation Index, *r*: Correlation coefficient, * *p* < 0.05.

## Data Availability

The data supporting the findings of this study are available from the corresponding author upon reasonable request.

## Abbreviations

The following abbreviations are used in this manuscript:

FBG	Fasting blood glucose
ACAT	Acyl-CoA cholesterol acyltransferase
NCEH1	Neutral cholesteryl ester hydrolase 1
ABCA1	ATP-binding cassette transporter A1
DPP-4i	dipeptidyl peptidase 4 inhibitors
GLP-1 RA	Glucagon-like peptide-1 receptor agonists
HbA1c	Glycated Hemoglobin
Neu	Neutrophils
Lym	Lymphocytes
PLT	Platelets
Mon	Monocytes
NLR	Neutrophil-to-Lymphocyte Ratio
LMR	Lymphocyte-to-Monocyte Ratio
PLR	Platelet-to-Lymphocyte Ratio
SII	Systemic Immune-Inflammation Index
